# Impacts of pharmacist-led medication reconciliation on discrepancies and 30-days post-discharge health services utilization in elderly Jordanians

**DOI:** 10.1371/journal.pone.0320699

**Published:** 2025-04-25

**Authors:** Eman A. Hammad, Farah Khaled, Majed Shafaamri, Bara’ah Amireh, Rasha Arabyat, Rana K. Abu-Farha

**Affiliations:** 1 Department of Biopharmaceutics and Clinical Pharmacy, School of Pharmacy, University of Jordan, Amman, Jordan; 2 Department of Health Economics and Healthcare Administration, Institute of Public Health, University of Jordan, Amman, Jordan; 3 Royal Medical Services, Queen Rania Pediatric Hospital, Total Parenteral Nutrition Unit, Amman, Jordan; 4 Clinical Pharmacy and Therapeutics Department, Faculty of Pharmacy, Applied Science Private University, Amman, Jordan; Isra University Faculty of Pharmacy, JORDAN

## Abstract

**Objectives:**

To assess the impacts of pharmacist-led medication reconciliation (MedRec) on medication discrepancies and post-discharge health services utilization in elderly patients in Jordan. And to identify predictors of post discharge outcomes.

**Method:**

Newly admitted patients, aged above 65 years were randomly allocated into either a group receiving pharmacist led MedRec or standard care. Within 24 hours of admission, a clinical pharmacist compiled a list of the Best Possible Medication History (BPMH) using at least two sources of information. The pharmacist compared the BPMHs to the admission charts to identify discrepancies and resolved them accordingly. One month post-discharge, patients were assessed for health services use, namely hospital readmissions, emergency department (ED) visits, and adverse drug events (ADEs). Logistic regressions used to investigate predictors of post discharge outcomes.

**Results:**

A total of 128 patients with 151 medication discrepancies were included: 82 (54.3%) discrepancies in the intervention group, and 69 (45.7%) in the control group. A total of 52 Pharmacist-led interventions were recommended to physicians, of which 49 (94.2%) were accepted/implemented. At discharge, the majority of unintentional discrepancies were successfully resolved (p < 0.001). At 30 days post-discharge, patients who were readmitted to the hospital and visited the ED were significantly from the control group. There was no significant difference with respect to experiencing ADEs among the study groups. Patients who received pharmacist-led MedRec had almost 70% lower likelihood of hospital readmission and ED visits. Discrepancies at discharge was associated with higher odds of hospital readmissions.

**Conclusion:**

Pharmacist-led MedRec services improved continuity of care for elderly patients. Implementing a structured reconciliation process successfully resolved discrepancies and reduced hospital readmissions as well as ED visits at 30-days post-discharge. This outlines potentials for healthcare cost savings. Future studies are recommended to explore long-term benefits, cost-effectiveness, and integrating pharmacist-led MedRec into standard discharge planning.

## Introduction

Medication discrepancies are defined as any difference between a patients’ current medications regimen and what is documented in their medical records, or prescribed across points of healthcare [1]. They commonly occur during care transitions, particularly in vulnerable populations such as geriatric patients [1–4]. This is often due to the involvement of multiple healthcare providers, medical comorbidities, and lack of structured information transfer processes [5,6]. Polypharmacy, cognitive and physiological difficulties may increase the likelihood of discrepancies mainly unintended drug omissions, discontinuations, duplications and formulation changes [3,4]. Research consistently shows that transitions between healthcare settings, such as hospital admissions or discharges, are very critical; because medication discrepancies can potentially occur, and remain unresolved [3,7,8]. This necessitates systematic approaches to ensure high accuracy, and completeness of patient information at points of health care transitions. Medication reconciliation (MedRec) has been identified as a potential intervention to improve care transitions and enhance patient safety. MedRec is defined as a formal process in which healthcare professionals systematically and accurately review patient’s medications list or information at every transition of care to ensure that all information are properly documented and accurate [9]. The involvement of pharmacists in MedRec was shown to contribute to patient safety and improving patient treatment outcomes across diseases and settings [2,5,8,10–12]. Focusing on patients at increased risk when implementing pharmacist led MedRec can optimize and inform effective implementation particularly in limited resources settings. Pharmacist-led transitional care program in elderly patients showed 26% lower risk of 30-day readmission, reduction in medication regimen complexity, decreased duplicated medications, unintentional discrepancies and reduced in-hospital medication costs across countries [13–16]. However, differences in healthcare systems, settings, and intervention designs across these studies highlight the need to study the effectiveness of pharmacist-led MedRec within the local context to assess its feasibility, impact, and potential for implementation. Involvement of pharmacist in MedRec services was associated with a significant reduction in medication discrepancies and improved quality of care in general medicine and surgical wards [17–19]. Geriatric populations remained understudied in Jordan and regionally [20]. Furthermore, the effects post-discharge health resources use including readmissions, and emergency department (ED) visits remain unclear [9,21]. Additionally, factors predicting optimal MedRec outcomes in elderly patients are not well understood, including patient-related factors, system or workflow process [16,20]. Thus, the aim of the study was to evaluate the effectiveness and feasibility of pharmacist-led MedRec services in elderly patients in Jordan on resolving medication discrepancies and preventing post discharge healthcare service utilization 30 days post-discharge. Additionally to identify factors influencing MedRec outcomes to provide insights into the integration of pharmacist-led MedRec into resource-limited settings.

## Method

### Study design, participants and clinical setting

A randomized, open labeled controlled trial was conducted between June and October 2018 at King Hussein Medical Hospital (KHMH), a leading Royal Medical Services (RMS) tertiary military hospital located in central of Amman, the capital of Jordan. The recruitment of study participants extended from June 24th, 2018, to September 13th, 2018. The clerk list of all hospitalized patients was obtained to determine newly admitted patients within 24 hours. Patients who were admitted during the weekends, and/or holidays were followed on the first working day. Admitted patients, aged above 65 years, and were prescribed at least one chronic medication prior to admission with anticipation to stay at hospital for more than 48 hours. Patients were excluded if they were admitted to the critical care or isolation units, unconscious or comatose, and were discharged against medical advice.

An information sheet outlining the study’s objectives, procedures, potential risks, and benefits was provided to all participants in Arabic language. Participants were given ample time to review the information and ask questions to ensure their understanding. Voluntary participation, with the option to withdraw from the study at any time without any consequences on their care was assured. Written informed consent was then obtained from each participant witnessed by the nurse in duty. Ethical approval for the study was obtained from the Institutional Review Board (IRB) of the RMS (Reference number: 6/2018). ClinicalTrials.gov registration identifier is NCT06610292.

### Randomization

Randomization was conducted using a simple randomization technique; participants were assigned to their groups based on a random number generator created using Microsoft Excel®. The randomization sequence was generated before recruitment and was kept concealed until allocation.

### Data collection

A standardized data collection form was designed for the study purpose. Medical files were reviewed to gather information related to age, gender, marital status, insurance type, date and reasons for admission, and previous medical histories. Moreover, information about the patients’ Best Possible Medication History (BPMH) was also extracted, including details on all medications taken prior to admission (both trade and generic names), dosages, frequencies, dosage forms, route and time of administrations, and start/stop dates. Information on current medications, both regular and as-needed, was also recorded. All data were cross-referenced with at least two reliable sources of information; the patients’ electronic records, and patient/caregiver interviews. For patients or caregivers unable to recall all medications; pre-admission medications charts, or discharge summaries were sought. Patients were also asked about any over-the-counter (OTC) medications, and herbal supplements use. The MedRec pharmacist followed up patients throughout their hospital stay, during any care transitions until discharge. This ensured the BPMH was continuously updated.

### Identification of medication discrepancies and potential clinical significance

The MedRec pharmacist compared patients’ current admission medications chart, and the BPMH list to identify any medication discrepancies. Discrepancies were categorized into: improper dosage, incorrect frequency, adding a new drug, duplication of drugs, or omission of previously used drug. Two authors (B.A and R.A) evaluated discrepancies to determine whether they were documented within patients’ medical records or not. All undocumented discrepancies were revised and deliberated with the care team to justify modifications on patients’ records. If the discrepancies were found to be justified, they were classified as intentional discrepancies (documentation errors). If not clarified; the discrepancies were reported as unintentional discrepancies. Unintentional discrepancies were defined as any medication discrepancies between patients’ medical records, inpatient medications charts, discharge summaries, and BPMHs with no identifiable rationale by responsible physician/s. All identified unintentional discrepancies were followed up to ensure that they were successfully resolved prior to discharge.

Unintentional discrepancies (medication errors) were classified into three classes based on their severity of potential harm, as described by Cornish and others [22]: “Class 1 discrepancies were those unlikely to cause patients discomfort or clinical deterioration. Class 2 discrepancies were those with the potential to cause moderate discomfort or clinical deterioration. Class 3 discrepancies had the potential to cause severe discomfort or clinical deterioration”. Classification of discrepancies was performed independently by two of the authors. Disagreements were resolved by discussions with an independent clinical pharmacist, until consensus was reached.

At discharge, medication discrepancies were assessed in both groups. Medication discrepancies identified upon discharge were discussed, and brought to the medical team’s attention before patients’ had left the hospital. Discharge summaries were then reviewed to ensure consistency of patients’ information.

### Pharmacists interventions

In the intervention group, the MedRec pharmacists documented medication discrepancies and sent a written consult note to the responsible physician/s. The intervention outcomes were recorded identifying whether they were accepted/ implemented or not.

Patients in the control group received standard care with no interference from the study pharmacists. Discrepancies were identified in the control group but not discussed with the healthcare team, unless life threatening and/or serious clinical deterioration was recognized. In this case, it was agreed to bring the discrepancies to the medical team’s attention, and exclude the patients from study analysis.

### Health resources utilization 30 days post-discharge

The time taken by the pharmacy team to deliver MedRec services was monitored. Additionally, for both study groups, the electronic medical records were accessed to gather data on re-hospitalizations, and ED visits within 30 days post-discharge. Then data were verified through phone calls with the patients.

Potential Adverse Drug Events (ADEs) were assessed by asking standardized screening questions regarding new or worsening medical symptoms since hospital discharge. In case of a confirmatory response on any question, a semi-structured interview was done to elicit a possible correlation with medications for each question [23].

### Statistical analysis

Data were entered and analyzed using Statistical Package for Social Science (SPSS) version 22 (SPSS Inc., Chicago, IL, USA). Descriptive analysis was done using means, and standard deviations (SD) for continuous variables, and percentages for categorical variables. Checking for normality was carried out using Shapiro-Wilk test, with p-value > 0.05 indicating normally distributed continuous variables.

Groups differences were conducted using independent sample t-test/Mann Whitney U test (depending on data normality), or by using Chi-Square/Fisher exact test when appropriate. Additionally, a paired t-test/Wilcoxon sign rank test was performed to ascertain whether pharmacists’ recommendations were effective in reducing the number of medication discrepancies. A p-value ≤ 0.05 was considered statistically significant, and all tests were two tailed. Exploratory models were built using logistic regressions to identify predictors of factors predicting better MedRec outcomes in elderly patients. Specifically, hospital readmission within 30 days and ED visits within 30 days. The dependent variables were: Hospital readmission within 30 days (binary: 1 =  readmitted, 0 =  not readmitted). ED visit within 30 days (binary: 1 =  ED visit, 0 =  no ED visit). The independent variables included 12 predictors reflecting patient characteristics, comorbidities, and medication factors. Variables were selected based on clinical relevance and prior research findings. Unadjusted and adjusted logistic regression analyses were conducted. Model Fit and Assumptions were assessed. Multicollinearity was assessed using the Variance Inflation Factor (VIF), with values > 10 considered problematic. Linearity of continuous predictors with the log-odds of the outcome was assessed using the Box-Tidwell test. The Hosmer-Lemeshow test was used to evaluate model goodness-of-fit. Sample size adequacy was ensured using the rule of at least 10 observations per predictor variable [24].

### Sample size calculation

To determine the required sample size, a power analysis with an alpha level of 0.05, and a power of 80% were set. With our aims toward piloting the feasibility, preliminary efficacy, as well as, post-discharge health sources savings. The sample size was calculated using G * Power 3.1. Based on an expected average effect size of 0.3 to 0.5 [17], the minimum required sample size was calculated to be 60 subjects per group [25].

## Results

### Demographic and medical characteristics of the study groups

**Fig 1:**
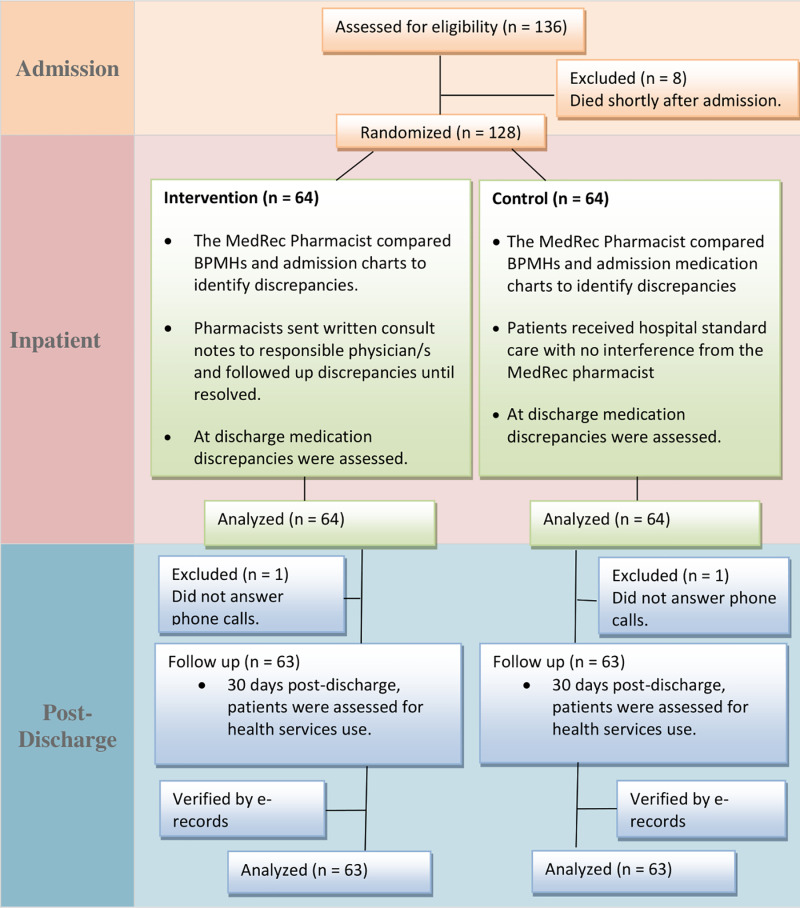
The flowchart of the study.

A total of 136 patients were screened for the study, eight patients were deceased before receiving MedRec services. None declined the study and thus 128 patients were randomized to the study groups. Table 1 summarizes the characteristics of the study groups. No significant differences were found between the two groups at baseline. (Fig 1) presents the study flowchart.

**Table 1:** Characteristics of the study sample per group (n = 64 per group).

	Measure	Intervention	Control
Age, years	Mean (SD)^*^	73.8 (5.50)	75.0 (7.66)
Gender	n (%)		
Male		39 (60.0%)	34 (53.1%)
Female		25 (40.0%)	30 (46.9%)
Marital Status	n (%)		
Married		68 (76.5%)	50 (78.1%)
Widowed		15 (23.4%)	14 (21.8%)
Educational levels	n (%)		
Not educated		19 (29.7%)	15 (23.5%)
Primary/high school		40 (62.5%)	41 (64.0%)
Diploma/BSc		05 (7.8%)	08 (12.5%)
Insurance	n (%)		
Military		53 (82.8%)	56 (87.7%)
Civilian		11 (17.1%)	08 (12.1%)
Number of Home Medications	Mean (SD)	6.82 (2.14)	6.81 (2.74)
Number of Current Medications	Mean (SD)	7.29 (2.34)	7.48 (2.88)
Number of Medical Conditions	Mean (SD)	2.87 (0.88)	2.84 (0.97)
Length of Stay (days)^*^	Mean (SD)	6.78 (5.30)	7.06 (5.69)
Admission Departments	n (%)		
Respiratory		06 (9.4%)	03 (4.7%)
Gastroenterology		02 (3.1%)	02 (3.1%)
Cardiology		17 (26.5%)	15 (23.4%)
Neurology		03 (4.7%)	10 (15.6%)
Nephrology/Urology		13(20.3%)	14 (21.9%)
Oncology/hematology		08 (12.5%)	10 (15.6%)
Infectious		04 (06.3%)	03 (4.7%)
Endocrinology		11 (17.1%)	07 (10.9%)
Reasons for admission,	n (%)		
Planned (e.g., Surgery)		08 (12.5%)	07 (11.0%)
Cancer (chemotherapy, radiation, workup, etc)		08 (12.5%)	08 (12.5%)
AKI		09 (14.1%)	10 (15.6%)
CAP or other infection		11 (17.2%)	12 (18.8%)
COPD exacerbation		01 (1.6%)	02 (3.1%)
ACS/Chest Pain		10 (15.6%)	11 (17.2%)
CKD		08 (12.5%)	08 (12.5%)
CVA (stroke, PE)		04 (6.3%)	03 (4.7%)
GI bleeding		03 (4.7%)	03 (4.7%)
Others		02 (3.1%)	02 (3.1%)

*LOS, reported in days, was calculated as the difference in time from arrival at the hospital and the time of discharge, as recorded in the hospital information system.

### Medication discrepancies and potential clinical significance

A total of 151 discrepancies were identified in the study groups; 82 (54.3%) discrepancies were identified in the intervention group, and 69 (45.7%) discrepancies in the control group. Fifty percent of patients had at least one unintentional discrepancy in both groups. Anti-hypertensive drugs (33.7%) were the most common drugs involved in medication discrepancies among the study groups, followed by anti-hyperlipidemia (9.5%), anti-diabetics (8.4%), anti-platelets, as well as, anti-epileptics (7.4%).

At admission, the average number of discrepancies was 1.28 (1.75) per patient in the intervention group, whilst 1.07 (1.13) per patient in the control group. This was not statistically significant; p = 0.47. Additionally, there was a total of 52 (34.4%) unintentional discrepancies deemed as medication errors averaged as 0.81 (1.23) per patient in the intervention group. The remainders were intentional discrepancies and deemed as documentation errors with an average 0.47 (1.34) per patient.

As for the control group; there were 43 (28.5%) unintentional discrepancies averaged as 0.67 (0.99) per patient. The remaining were intentional discrepancies, and averaged as 0.41 (1.00) per patient.

**Fig 2:**
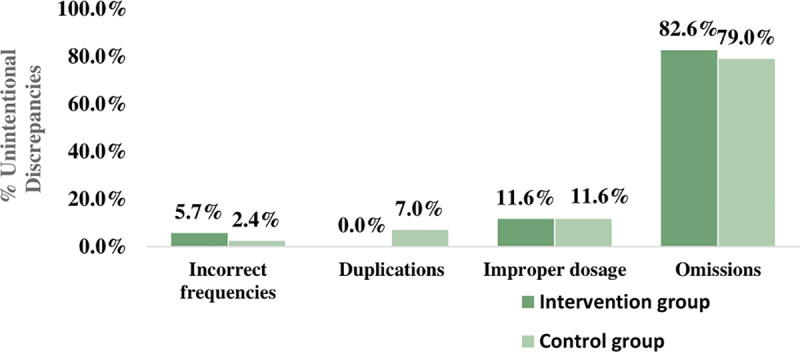
Types and frequencies of identified unintentional discrepancies for both groups.

Fig 2 summarizes the most common types of identified unintentional discrepancies for both groups. The most common discrepancies were omissions, followed by incorrect dosages for both groups too.

For both groups, most unintentional discrepancies were classified as moderate to severe potentially harmful discrepancies, “Class 2” and “Class 3”; 50 (96.1%) in the intervention group, and 40 (93%) or the control group. Table 2 represents examples of medication discrepancies, pharmacists’ interventions, and potential clinical significance.

**Table 2:** Examples of the identified unintentional medication discrepancies and potentail clinical signifcance.

Types of discrepancies	Details of discrepancies	Pharmacist-led MedRec interventions	Potential clinical signifcance [22]
Omission	Patient was on Omeprazole 20 mg (Losec^®^) once daily, the resident did not order it on admission.	The MedRec pharmacist contacted the resident and the attending physician to inform them about the omission, and clarified why Omeprazole should be continued for stress ulcer prophylaxis. Taking into consideration that there was no Drug-Drug interactions.	Class 1
Duplication	Patient was on Sertraline 50 mg (Zoloft^®^) once daily. At admission the resident prescribed Citalopram 20 mg (Cipram^®^) once daily. Both medications were charted to the patient during hospital stay.	The MedRec Pharmacist confirmed the patient’s prior use of Sertraline, duration of therapy, and verified no previous issues with efficacy, and side effects. Duplication was stopped.	Class 2
Omission	Patient was on Warfarin 5 mg (Orfarin^®^) once daily; the resident did not order it on admission	The MedRec Pharmacist immediately contacted the resident and the attending physician to inform them about the omission, and clarified why Warfarin should be reinitiated. The INR Level was checked to assess the coagulation status, and risks of thromboembolic events.	Class 3
Improper dosage	Patient was on Isosorbide dinitrate 10 mg (Isoket^®^) twice daily; on admission the order was Isoket^®^ 20 mg twice daily.	The MedRec Pharmacist checked the patient’s previous medication records to confirm the correct dose, and consulted the prescriber if increasing dose was intentional. Also, checked if the patient experienced any side effects (e.g., headaches, dizziness, or hypotension)	Class 3
Incorrect frequency	Patient on Phenytoin 100 mg (Epanutin^®^) twice daily. At admission the order was once daily, without justification.	The MedRec Pharmacist contacted the resident to clarified whether the change was intentional, such as a change in the patient’s condition (e.g., reduced seizure frequency, or potential drug interactions), and emphasized maintaining therapeutic levels.	Class 3

MedRec = Medication reconciliation, INR = International normalized ratio.

The study pharmacists recommended interventions to resolve the 52 unintentional discrepancies in the intervention group, 49 (94.2%) of these recommendations were accepted by responsible physician/s. Among accepted recommendations, 45 (86.5%) recommendations were successfully resolved. Only 3 (5.7%) recommendations were rejected by physicians due to clinical judgment.

No interventions were implemented for the control group and when compared to the intervention group, there was a statistically significant reduction in the number of unintentional discrepancies in the intervention group. Of 69 (45.7%) discrepancies identified for the control group, 42 (60.9%) discrepancies were resumed at discharge. Of which 30 (75.0%) discrepancies were unintentional discrepancies.

### Health resources utilization and ADEs within 30 days of discharge

The time taken by the pharmacists to implement MedRec services to the intervention group was 54.1 (31.99) min per patient (range 20–180 min).

Only two (1.6%) patients were lost to follow up due to not answering phone calls and thus 63 patients in each group were assessed for post-discharge health resources use (Fig 1). At 30 days post-discharge, 18 (28.6%) patients were readmitted in the intervention group compared to 30 (47.6%) patients in the control group (p = 0.028). As for ED visits, among intervention group 16 (25.4%) patients visited the ED compared to 28 (44.4%) patients in the control group (p = 0.026). On the other hand, 14 (22.2%) patients in the control group reported ADEs such as uncontrolled blood pressure, hyper- or hypoglycemia, or low heart rate compared to 10 (15.9%) patients in the intervention group (p = 0.37).

### Factors predicting post discharge outcomes in elderly patients: Exploratory models

A total of 128 patients were included in the analysis, with 48 patients (37.5%) experiencing hospital readmission within 30 days and 44 (34%) experiencing an ED visit within 30 days. Table 3 presents the results of both unadjusted and adjusted logistic regression models for hospital readmission and ED visits.

**Table 3:** The results unadjusted and adjusted logistic regression models for hospital readmission and ED visits.

	Hospital readmission within 30 days						ED visit within 30 days					
	Unadjusted			Adjusted			Unadjusted			Adjusted		
	OR	95%CI	p	OR	95%CI	p	OR	95%CI	p	OR	95%CI	p
Received Pharmacist led MedRec (Y = 1)	0.25	[0.07–0.85]	**0.03**	0.31	[0.13–73]	**0.01**	0.2	[0.06–0.65]	**0.01**	0.32	[0.14–0.74]	**0.01**
Gender (M = 1, F = 2)	0.8	[0.32–2.02]	0.64	0.82	[0.33–2.03]	0.67	0.67	[0.26–1.69]	0.39	0.7	[0.28–1.75]	0.45
Age (years)	0.93	[0.86–1.01]	**0.08**	0.94	[0.87–1.01]	0.10	0.9	[0.83–0.98]	**0.02**	0.91	[0.84–0.99]	**0.02**
Marital status	1.37	[0.77–2.45]	0.29	1.41	[0.81–2.45]	0.23	0.97	[0.53–1.75]	0.91	0.96	[0.54–1.71]	0.89
Education level (0 = No education; 3:BSc)	0.47	[0.19–1.16]	0.1	0.54	[0.23–1.25]	0.15	0.39	[0.15–0.98]	0.05	0.46	[0.19–1.07]	**0.07**
No. home medications	1.18	[0.99–1.41]	**0.06**	1.17	[0.99–1.41]	**0.07**	1.04	[0.8–1.36]	0.75	1.01	[0.82–124]	0.92
Admitting specialty	0.9	[0.69–1.17]	0.44	0.88	[0.71–1.08]	0.22	0.97	[0.81–1.16]	0.71	0.95	[0.8–1.14]	0.59
No. comorbidities	1.59	[0.94–2.69]	**0.08**	1.64	[0.98–2.67]	**0.06**	1.33	[0.79–2.24]	0.29	1.36	[0.82–2.27]	0.24
Total number of discrepancies	0.91	[0.59–1.41]	0.67	0.96	[0.64–1.44]	0.86	0.86	[0.55–1.33]	0.49	0.91	[0.61–1.37]	0.65
At least one unintentional discrepancy	1.18	[0.31–4.52]	0.81	1.39	[0.41–4.68]	0.6	1.64	[0.43–6.21]	0.46	1.69	[0.5–5.7]	0.40
Length of stay	0.97	[0.9–1.05]	0.45	0.98	[0.91–1.05]	0.54	0.96	[0.88–1.04]	0.29	0.97	[0.9–1.05]	0.44
No. of discrepancies at discharge	1.58	[1.13–2.19]	**0.01**	1.5	[1.04–2.16]	**0.03**	1.25	[0.91–1.73]	0.17	1.33	[0.99–1.78]	**0.06**

OR: Odds Ratio; CI: Confidence Interval; ED: Emergency Department, MedRec: Medication Reconciliation.

For readmission, patients received pharmacy-led MedRec group had lower odds of readmission. Increased number of discrepancies at discharge was a significant predictor of readmission. Increased number of home medications and co-morbidities increased the likelihood of readmission within 30 days, however, this was not statistically significant.

Patients in the intervention group were less likely to visit the ED too. Older patients were at lower odds for visiting ED. Higher Educational level predicted lower odds for visiting ED whilst increased number of discrepancies at discharge increased odds for visiting ED. However, this was almost statistically significant.

Multicollinearity was not detected, as all VIF were below 3. The Box-Tidwell test indicated no significant violations of linearity for continuous variables. The Hosmer-Lemeshow test confirmed a good model fit (p = 0.97 and 0.41) for both models respectively.

## Discussion

The study demonstrated significant impacts of pharmacist-led MedRec services on resolving medication discrepancies during hospital admissions. Medication discrepancies were prevalent in elderly patients, approximately 50% of patients in each group experienced at least one unintentional discrepancy upon admission. The most common type of discrepancies was omissions, followed by improper dosages. Anti-hypertensive drugs were the most commonly implicated class of medications. These findings are consistent with previous studies emphasizing the vulnerability of older adults to medication discrepancies, and the roles of MedRec services in the resolution of these discrepancies across healthcare transitions [9,26,27]. In addition to resolving discrepancies, pharmacist-led MedRec services had positive effects on post-discharge health sources use contributing to fewer hospital readmissions, and ED visits within 30 days post-discharge. This is in line with previous studies too [1,2,17,28,29]. Hospital readmissions, and ED visits contribute to substantial healthcare costs [3,28,30]. Thus, the pharmacist-led MedRec services could contribute to overall healthcare cost-savings [14,16,31]. Yet, further systematic investigation of costs and outcomes is needed to inform resources needed and cost-effectiveness.

In Jordan, the implementation and standardization of MedRec services are still developing. While there is a growing recognition of its importance, MedRec as a process is not fully established and vary across settings and organizational layouts [9,17,32]. Studies outlined readiness among healthcare teams to collaborate with pharmacists to provide MedRec services. Yet, the lack of standardized procedures and insufficient training, and time constraints were found major barriers to the standardize implementation of MedRec services [32]. Nonetheless, the average time required for MedRec services in this study was approximately 54.1 minutes per patient, and was comparable to previous reports [3,7]. This is a considerable investment in time, particularly in high workload settings, or when time constraints exist. However, the time commitment by the pharmacist can be overweighed by the benefits on health sources saving [4,6,29,31]. Furthermore, strategies that can enhance feasibility in high-workload environments can be advocated. Strategies involving trained allied staff such as pharmacy technicians in data collection, verification, and initial medication reviews can potentially enhance feasibility [20]. Utilizing electronic health records and automated MedRec tools can also optimize implementation in these settings [14,28]. Prioritize MedRec efforts on high-risk patients or complex care regimens, responsibilities sharing relaying on interdisciplinary approach for MedRec can enhance feasibility too [2,16,20].

The exploratory models for factors predicting better outcomes showed that receiving pharmacist-led MedRec was significantly associated with lower odds of hospital readmission and ED visits in both unadjusted and adjusted models. Patients who received pharmacist-led MedRec had almost 70% lower likelihood of hospital readmission and ED visits. Having more discrepancies at discharge was associated with higher odds of hospital readmission. However, this variable did not reach statistical significance for ED visits (p =  0.06). Overall, these findings highlight the protective role of pharmacist-led MedRec in reducing hospital readmissions and ED visits [28,33]. Other demographics and clinical information including number of home medications, number of comorbidities, and length of stay were not significant predictors. This is in disagreement with a comparative retrospective study of risk factors for hospital readmission in older adults within 30 days of discharge in Sweden and a 3 year a retrospective analysis in Canada [7,30]. This might indicate that pharmacist-led interventions, such as MedRec, may mitigate the impacts of polypharmacy and comorbidities on post-discharge outcomes. This need to be further evaluated in larger future studies.

Worth noting, medication discrepancies at discharge shown to contribute to increased readmission risk. Unresolved discrepancies, particularly at discharge involving high-risk medications would increase the risk of medication-related hospitalizations and treatment failures [6,8,10]. Future studies should explore strategies to integrate MedRec into discharge planning and assess its cost-effectiveness in various healthcare settings.

Balancing the feasibility of implementing MedRec services with its cost-saving potential is crucial for healthcare organizations. The broader implications of MedRec services, particularly in enhancing patient safety, and preventing costly hospital readmissions and/or ED visits should be considered a significant driver to seek MedRec processes despite its initial setups, and resources allocation including trained personnels, technology integrations, and workflow organizations [34,35]. However, large scale studies to evaluate organizational costs and net costs saving are needed.

### Study strengths and limitations

This study provides focused insights on feasibility and effectiveness of the pharmacy led services on elderly patients, a population particularly vulnerable to medication errors and discrepancies [7,16,21]. In addition, this study explores key factors influencing hospital readmission and ED visits and provides valuable insights for integrating medication pharmacist-led MedRec services into discharge planning and transitional care strategies. However, the study was conducted in a single site and was based on military specialized center. Thus, the layout, organizational care for study population and care processes vary and thus generalizability might not be warranted to other civilian or non-specialized hospitals.

Additionally, MedRec services were performed by a single pharmacist, which introduced a potential for observer’s bias. Expanding the scope in future studies to include multiple pharmacists and healthcare settings can allow for better investigation of variability in practices and outcomes across different settings.

Further to this, the assessment of the potential clinical significance of medication discrepancies was performed by the pharmacists involved in the MedRec interventions. Independent assessment would have minimized measurement bias and provided a more objective evaluation of the potential clinical significance. However, this study aimed to assess the feasibility of pharmacist-led MedRec services and to provide preliminary data to inform future costing and cost-effectiveness studies. This feasibility study provides valuable insights into the time and resources required for MedRec services implementation. It can also inform future larger-scale trials evaluating cost-effectiveness and resources needed for optimum implementation.

## Conclusion

Pharmacist-led MedRec services significantly reduced medication discrepancies and improved post-discharge outcomes among elderly patients. Implementing structured MedRec processes during hospital admissions and discharges can enhance patient safety, reduce health services use, and potentially lower healthcare costs. Future research should focus on optimizing MedRec workflows and expanding these services to a broader range of healthcare settings. Although time constraints pose challenges for MedRec implementation, the potentials for cost savings might improve healthcare efficiency.

## Supporting information

S1 File Data set supplementary information. (SAV)
